# Nuclear Factor-κB Activating Protein Plays an Oncogenic Role in Neuroblastoma Tumorigenesis and Recurrence Through the Phosphatidylinositol 3-Kinase/Protein Kinase B Signaling Pathway

**DOI:** 10.3389/fcell.2020.622793

**Published:** 2021-01-21

**Authors:** Jun Liu, Mingyu Zhang, Ying Kan, Wei Wang, Jie Liu, Jianhua Gong, Jigang Yang

**Affiliations:** ^1^Department of Nuclear Medicine, Beijing Friendship Hospital, Affiliated to Capital Medical University, Beijing, China; ^2^Oncology Department, Institute of Medicinal Biotechnology, Chinese Academy of Medical Sciences & Peking Union Medical College, Beijing, China

**Keywords:** neuroblastoma, tumorigenesis, recurrence, nuclear factor-κB activating protein, phosphatidylinositol 3-kinase/protein kinase B signaling pathway

## Abstract

Nuclear factor-κB activating protein (NKAP) is a conserved nuclear protein that acts as an oncogene in various cancers and is associated with a poor prognosis. This study aimed to investigate the role of NKAP in neuroblastoma (NB) progression and recurrence. We compared NKAP gene expression between 89 recurrence and 134 non-recurrence patients with NB by utilizing the ArrayExpress database. The relationship between NKAP expression and clinicopathological features was evaluated by correlation analysis. We knocked down NKAP expression in NB1 and SK-N-SH cells by small interfering RNA transfection to verify its role in tumor proliferation, apoptosis, and the phosphatidylinositol 3-kinase/protein kinase B (PI3K/AKT) signaling pathway. NKAP gene expression in NB tissues was significantly overexpressed in the recurrence group compared with the non-recurrence group, and NKAP was enriched in the PI3K/AKT pathway. Correlation analysis revealed NKAP expression was correlated with chromosome 11q deletion in patients with NB. Knockdown of NKAP expression significantly inhibited the proliferation and promoted the apoptosis of NB1 and SK-N-SH cells. Moreover, we found that small interfering NKAP significantly reduced p-PI3K and p-AKT expression. NKAP knockdown played an oncogenic role in NB by inhibiting PI3K/AKT signaling pathway activations both *in vitro* and *in vivo*. Our research revealed that NKAP mediates NB cells by inhibited proliferation and promoted apoptosis through activating the PI3K/AKT signaling pathways, and the expression of NKAP may act as a novel biomarker for predicting recurrence and chromosome 11q deletion in patients with NB.

## Introduction

Neuroblastoma (NB) is a malignant embryonal tumor of neural crest cells and is the most common tumor in the less-than-1-year-old pediatric population (Heck et al., 2009; Salazar et al., 2016). Neural crest cells are a group of transient and multipotent cells that are induced to migrate and differentiate. However, under certain conditions, neural crest cells with defects relating to migration, maturation, or differentiation can result in the development of NB along the sympathetic nervous system most frequently in the adrenal glands followed by the abdomen or pelvis (Ishola and Chung, 2007; Whittle et al., 2017). Treatment of NB is based on risk stratification, including surgery, chemotherapy, radiotherapy, and immunotherapy. Prevention of tumor recurrence is particularly difficult in high-risk NB patients, and the 5-year survival rate of patients with NB who experience recurrence is less than 50% (Cheung et al., 2015; DuBois et al., 2017). 11q deletion is known to be a recurrent genetic alteration and a dismal prognostic factor in NB. Its presence predicts a bad outcome and increases relapse probability, especially in localized stages and 4s stages (Juan Ribelles et al., 2019). Currently, the most common methods for detecting recurrence and metastatic lesions are imaging methods and cytological examinations. However, by the time these diagnostic methods are used, the tumor usually progresses to a more advanced stage. Therefore, there is a considerable need to identify novel and effective biomarkers to predict NB recurrence (Su et al., 2020). The prognosis of patients with markedly NB varies owing to NB heterogeneity. Some children with NB can experience spontaneous regression or differentiation into a benign ganglioneuroma without treatment, but others experience widespread metastasis with poor outcomes despite aggressive multimodal therapy (Brodeur, 2018). NB is a challengingly complex disease with unpredictable tumor progression. Molecular mechanism analysis contributes to the understanding of the biological behavior of NB and is crucial for identifying new biomarkers to predict recurrence, clinicopathological features, and also provide new targets to optimize treatment strategies for NB.

Nuclear factor-κB (NF-κB) is a transcription factor found in essentially all cell types and regulates the transcription of numerous genes involved in infection, inflammation, immunity, and cancer (Karin and Ben-Neriah, 2000). NF-κB activating protein (NKAP) is a novel nuclear regulator of NF-κB activation (Chen et al., 2003). Previous studies show that NKAP functions as a negative regulator for T cells, invariant natural killer T cells (iNKT), and regulatory T cells in maturation and the acquisition of functional competency through Notch signaling (Hsu et al., 2011; Thapa et al., 2013). NKAP also acts as an essential transcriptional regulator for adult hematopoietic stem cells maintenance and survival (Pajerowski et al., 2010). In addition, NKAP functions as an oncogene in breast cancer, hepatocellular carcinoma, and renal cell carcinoma through the protein kinase B/mammalian target of the rapamycin signaling pathway (Liu et al., 2018; Song et al., 2019; Ma et al., 2020). However, the function of NKAP in NB cells has not yet been elucidated.

In this study, we aimed to explore the role of NKAP in the progression of NB and its correlation with clinicopathological features in pediatric patients with NB. We found that NKAP mediated the proliferation and apoptosis of human NB cells through activating the phosphatidylinositol 3-kinase (PI3K)/AKT signaling pathways, and the expression of NKAP may act as a novel biomarker for predicting recurrence and chromosome 11q deletion in patients with NB.

## Materials and Methods

### Bioinformatics Analysis

The NB dataset, which consisted of 89 recurrence and 134 non-recurrence NB tissues, was obtained from the ArrayExpress database^1^. The transcripts per million reads data of NKAP were used to compare the expression between recurrence and non-recurrence NB tissues. To determine the enrichment score of specific signatures in the gene sets positively correlated with NKAP expression in the NB profile, a gene set enrichment analysis (GSEA v2.2.3)^2^ was performed. Samples from the NB dataset in this study were divided into high- or low-NKAP expression groups using the median as the cutoff. The canonical pathways gene sets from the Molecular Signatures Database (MsigDB)^3^ were used for enrichment analysis. Default settings were used, and thresholds for significance were determined by permutation analysis (1,000 permutations). The false discovery rate was calculated. A function or pathway term with an adjusted *P* < 0.05 and false discovery rate *q* < 0.25 [(normalized enrichment score) > 1] was considered to indicate statistically significant enrichment.

### Tissue Specimens

A total of 34 specimens were obtained from children with NB who underwent surgical resection at Beijing Children’s Hospital, Capital Medical University, from March 2018 to August 2019. NB specimens were prepared as paraffin sections and primed for immunohistochemistry (IHC) staining. The Research Ethics Committee of Beijing Friendship Hospital, Capital Medical University (institutional review board: 2018-P2-145-02), approved this study, and all the legal guardians of child patients provided written informed consent.

### Cell Culture

The human NB cell lines IMR-32, NB1, SK-N-SH, and SK-N-BE2 and the human embryonic kidney293T cell line were purchased from the American Type Culture Collection (United States). All cell lines were maintained in high glucose Dulbecco’s modified Eagle medium (Hyclone, United States) supplemented with 10% fetal bovine serum (Gibco Corporation, United States) and 1% penicillin–streptomycin (Solarbio Science & Technology, China). All cells were cultured in a humidified atmosphere containing 5% carbon dioxide at 37°C.

### Xenograft Model Preparation

Six-week-old BALB/c nude mice (weight: 16–18 g) were purchased from Beijing Vital River Laboratory Animal Technology Co. (Beijing, China). All animals were housed at a temperature of 22 ± 2°C, relative humidity of 55 ± 10%, and under a light/dark cycle of 12/12 h. After transfected with small interfering NKAP-1 (siNKAP-1) or small interfering negative control (siNC) lentivirus, SK-N-SH cells (5 × 10^6^) were injected subcutaneously into the mice (*n* = 6 for siNKAP-1 and siNC, respectively). Tumor growth was monitored every 3 days after injection using a standard caliper. Tumor volume (cubic millimeter) was calculated as follows: 1/2 (length × width^2^). At the end of the experiments, the mice were euthanized, and tumor tissues were collected, weighed, and processed for IHC. All animal experiments were in compliance with the protocols approved by the Institutional Animal Care and Use Committee of the Institute of Medicinal Biotechnology, Chinese Academy of Medical Sciences (IMBF20200301).

### Lentivirus Transduction

The expression of NKAP in the NB1 and SK-N-SH cell lines was knocked down using lentivirus transduction. The lentiviral particles were produced by transfecting HEK293T cells with the lentiviral plasmids. Cells at the logarithmic phase were collected and inoculated into a six-well plate (Corning Costar, Corning, NY, United States) at a density of 2 × 10^5^ cells/well. siNKAP or siNC for transfection into NB1 and SK-N-SH cells following the manufacturer’s instructions. After 72 h of incubation, NKAP expression was determined using quantitative PCR and Western blotting.

### Quantitative Real-Time PCR Assay

The messenger RNA (mRNA) expression of NKAP genes was detected by quantitative real-time PCR. Total RNA was extracted from cells and tissues using Trizol reagent and reverse-transcribed into complementary DNA using a RevertAid First Strand cDNA Synthesis Kit (Fermentas, United States). NKAP gene expression was examined using quantitative real-time PCR through an ABI Prism 7300 Fast Real-Time PCR System (Applied Biosystems, United States) and SYBR Green PCR kit (Thermo Fisher Scientific, United States). The primer sequences were as follows: NKAP, forward, 5′ CCGAAGCCCAGCAAATC 3′ and reverse, 5′ AGGAGGCAG AAGCGAAGG 3′; glyceraldehyde-3-phosphate dehydrogenase, forward, 5′ AATCCCATCACCATCTTC 3′ and reverse, 5′ AGGCTGTTGTCATACTTC 3′. The results were normalized to the level of glyceraldehyde-3-phosphate dehydrogenase.

### Western Blot Assay

The protein expression of NKAP was analyzed by Western blotting. Cells were lysed in ice-cold radioimmunoprecipitation assay buffer for 30 min at 4°C. Protein concentrations were determined using a bicinchoninic acid protein assay kit (Thermo Fisher Scientific, United States). Equivalent amounts of protein were resolved in 10 or 12% sodium dodecyl sulfate polyacrylamide gel electrophoresis gel and transferred to polyvinylidene fluoride membranes (Millipore). The membranes were blocked with 10% non-fat dry milk in Tris-buffered saline containing 0.1% Tween-20 for 1 h and then incubated overnight at 4°C with the following antibodies: anti-NKAP (1:1,000; Abcam, Ab229096), anti-PCNA (1:2,000; Abcam, Ab152112), anti-Cyclin D1 (1:2,000; Abcam, Ab16663), anti-Bcl-2 (1:1,000; Abcam, Ab59348), anti-Bax (1:1,000; Abcam, Ab182734), anti-Caspase3 (1:1,000; Abcam, Ab90437), anti-PI3K (1:1,000; Abcam, Ab32089), anti-P-PI3K (1:1,000; CST, #13857), anti-AKT (1:1,000; CST, #4691), anti-P-AKT (1:2,000; CST, #4060), and anti-glyceraldehyde-3-phosphate dehydrogenase (1:2,000; CST, #5174), followed by additional incubation with horseradish peroxidase-conjugated goat anti-rabbit secondary antibody (1:5,000; Beyotime Biotechnology, China, A0208) for 1 h at room temperature. The bands were visualized using a Tanon-5200 Chemiluminescent Imaging system (Tanon Science & Technology, China), and Image J software (NIH) was used to quantify the expression of target proteins by normalization to glyceraldehyde-3-phosphate dehydrogenase.

### Immunohistochemistry Staining

The tumor specimens were fixed in 10% formalin for 48 h, embedded in paraffin, and cut into 3-μm-thick sections. IHC was performed as previously described (Ding et al., 2010). Briefly, the slides were incubated with anti-NKAP (1:100, Abcam) at 4°C overnight. Next, the slides were incubated with horseradish peroxidase-labeled goat anti-mouse or anti-rabbit secondary antibody (Boster, Wuhan, China) at room temperature, followed by counterstaining with hematoxylin. The staining was observed under a BX53 Olympus microscope (Olympus, Japan) at a magnification of 200×. Brown-yellow staining was defined as positive expression. NKAP protein was quantitated by Image-J software (NIH, Bethesda, MD, United States).

### Cell Counting Kit-8 Assay

NB1 and SK-N-SH cell viability was examined using a Cell Counting Kit (CCK-8) (Signalway Antibody; United States) assay according to the manufacturer’s instructions. Approximately 3,000 cells/well were seeded onto 96-well plates and cultured overnight with 100 μl of the complete medium. After incubation for 0, 24, 48, and 72 h, the contents of each well were added to 100 μl of fresh medium containing 10 μl of CCK-8 reagent. Incubation lasted for 2 h at 37°C. The absorbance at a 450 nm wavelength was detected using an Infinite 200 Pro microplate reader.

### Apoptosis Assay

Cells were digested with ethylenediaminetetraacetic acid-free trypsin and centrifuged at 1,500 rpm for 5 min. The cells (1 × 10^6^) were then washed with cold phosphate-buffered saline and resuspended in 1 × binding buffer containing 5-μl Annexin V-FITC (Beyotime Biotechnology, China) and 5-μl propidium iodide for 15 min at 4°C. The proportion of apoptotic cells was detected by flow cytometry (FACS Calibur, Becton Dickinson, United States), and data were analyzed by FlowJo software.

### Statistical Analysis

All data are expressed as the mean ± standard deviation or frequency. Statistical analysis was performed using SPSS software (version 22.0, IBM, Armonk, NY, United States). The differences between groups were analyzed using an independent sample test and one-way analysis of variance with a least-significant difference or Tamhane test. The difference in optical density values at different time points was analyzed using analysis of variance for repeated measurements. The correlation between NKAP expression and clinicopathological features in children with NB was analyzed using Pearson’s and Spearman’s correlation coefficients. *P* < 0.05 was considered statistically significant.

## Results

### Expression of Nuclear Factor-κB Activating Protein in Children With Neuroblastoma and the Correlation Between Nuclear Factor-κB Activating Protein and Clinicopathological Characteristics

**FIGURE 1:**
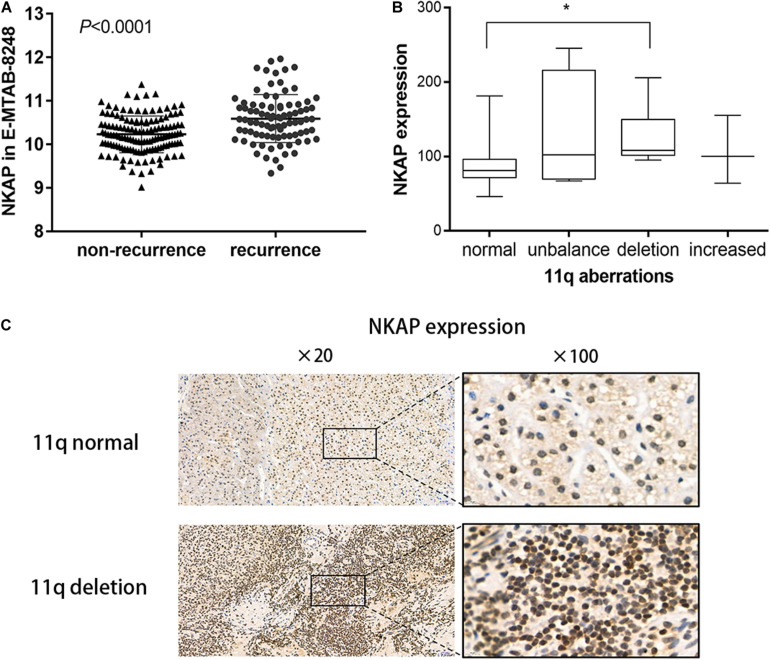
Expression of NKAP in human neuroblastoma tissue. **(A)** Difference of NKAP gene between non-relapse group and relapse group of NB tissues. **(B)** Box figure of NKAP expression among different 11q aberration patterns. 11q normal and 11q deletion show a statistical difference in NKAP expression. **(C)** IHC analysis of NB children with tissue 11q normal and 11q deletion. *P < 0.05.

We analyzed the differences in NKAP gene expression for 89 recurrence and 134 non-recurrence NB tissues using the ArrayExpress database. The NKAP gene in NB tissues was significantly overexpressed in the recurrence group compared with the non-recurrence group (*P* < 0.0001) (Figure 1A). We subsequently performed an IHC assay to investigate the relationship between NKAP expression and clinicopathological features in children with NB. We found that NKAP expression was correlated with chromosome 11q deletion in NB tissues (*r* = 0.42, *P* = 0.013). The expression of NKAP in children with NB with chromosome 11q deletion was significantly higher than in those with normal chromosome 11q (*P* < 0.05) (Figures 1B,C).

### Expression of Nuclear Factor-κB Activating Protein in Various Human Neuroblastoma Cell Lines and Decreased After Small Interfering Nuclear Factor-κB Activating Protein Transfection

**FIGURE 2:**
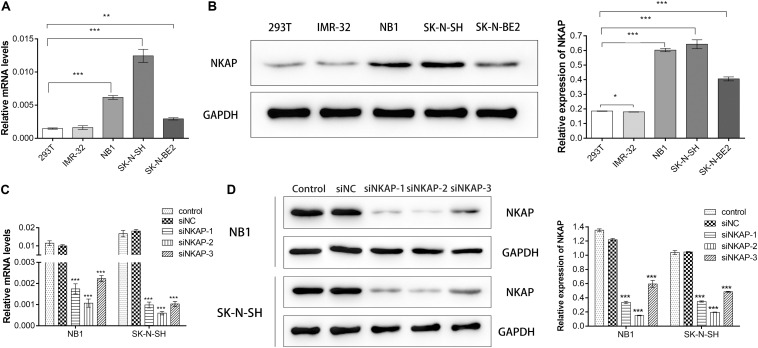
Expression of NKAP in neuroblastoma cell lines and verify of knockdown efficiency of NKAP. NB1 and SK-N-SH cells were transfected with siNKAP-1, 2, and 3. Non-specific lentiviral vectors with non-specific were used as negative controls (siNC). **(A)** mRNA levels of NKAP were detected by quantitative real-time PCR. **(B)** Protein levels of NKAP were detected by Western blot. **(C)** Quantitative real-time PCR assay was performed to test knockdown efficiency of NKAP. **(D)** Western blot assay was performed to test knockdown efficiency of NKAP. All data were expressed as mean ± standard deviation of three independent experiments. ***P < 0.001, **P < 0.01, *P < 0.05.

We detected NKAP expression in 293T, IMR-32, NB1, SK-N-SH, and SK-N-BE2 cells at the mRNA and protein levels. 293T cell is a normal cell line of human renal endothelial used for normal control. NKAP expression was higher in NB1 and SK-N-SH cell lines than in others at both the mRNA and protein levels (Figures 2A,B). To investigate the function of NKAP in NB progression, NB1 and SK-N-SH cells were transfected with siNKAP plasmid to downregulate its expression. The results of this knockdown indicated that the expression of NKAP significantly decreased at both the mRNA and protein levels after transfection with siRNA (Figures 2C,D). siRNA-1 and siRNA-2 demonstrated preponderant activity in knocking down NKAP expression and were selected for follow-up experiments.

### Knockdown of Nuclear Factor-κB Activating Protein Inhibited the Proliferation of NB1 and SK-N-SH Cells

**FIGURE 3:**
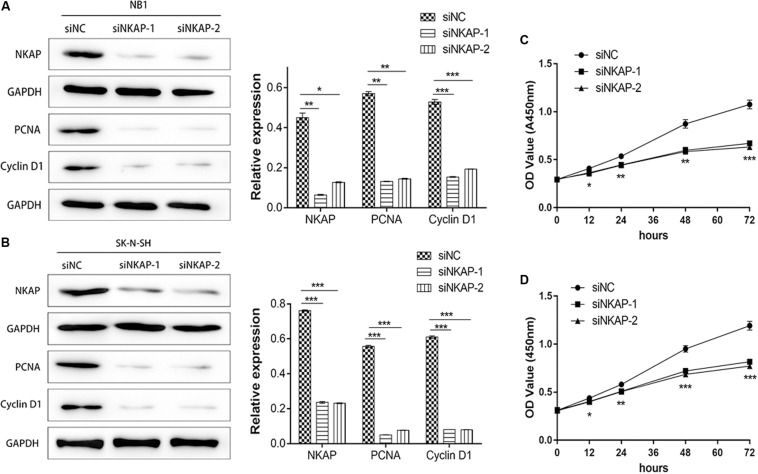
Knockdown of NKAP attenuated proliferation of neuroblastoma cells *in vitro* and *in vivo*. Western blot showed that siNKAP inhibited proliferation of NB1 cell **(A)** and SK-N-SH cell **(B)** compared with siNC. Cell proliferation of NB1 **(C)** and SK-N-SH **(D)** detected by CCK8 assay. ***P < 0.001, **P < 0.01, *P < 0.05.

Accelerated proliferation is one of the essential characteristics of neoplastic cells. Therefore, we investigated the role of NKAP in the tumor proliferation of NB cells *in vitro*. Tumor proliferation-associated proteins of PCNA and Cyclin D1 were detected by Western blot assay, whereas cell proliferation was detected by CCK-8 assay. A significant decrease in the expression of NKAP, PCNA, and Cyclin D1 in NB1 and SK-N-SH cells was detected after transfection with siNKAP compared with siNC cells (*P* < 0.05) (Figures 3A,B). The proliferation of NB1 and SK-N-SH cells transfected with siNKAP was also significantly inhibited compared with siNC cells (*P* < 0.001) (Figures 3C,D).

### Knockdown of Nuclear Factor-κB Activating Protein Promoted the Apoptosis of NB1 and SK-N-SH Cells

**FIGURE 4:**
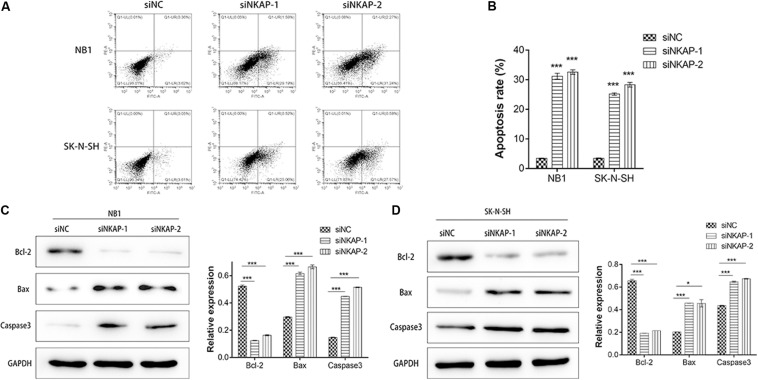
Knockdown of NKAP promotes apoptosis of neuroblastoma cells. **(A,B)** Effect of NKAP low expression on apoptosis in NB1 and SK-N-SH cells was examined by flow cytometry assay using an Annexin V-FITC Apoptosis Detection Kit. Apoptosis-associated proteins of Bcl-2, Bax, and Caspase3 were examined by Western blot in NB1 cells **(C)** and SK-N-SH cells **(D)** after transfected with siNC, siNKAP-1, and siNKAP-2. ***P < 0.001, *P < 0.05.

Next, we investigated the impact of NKAP on NB1 and SK-N-SH cells apoptosis. Cell apoptosis was analyzed using a flow cytometry assay, and apoptosis-associated proteins of Bcl-2, Bax, and Caspase3 were detected by Western blot assay. The apoptosis percentage of siNKAP transfected NB1 and SK-N-SH cells was significantly higher than that of the siNC group (*P* < 0.001) (Figures 4A,B). Western blotting showed that siNKAP transfection promoted the expression of Bax and Caspase3 protein and reduced the expression of Bcl-2 (Figures 4C,D). This suggested that NKAP could affect the cellular apoptosis of NB, as knockdown of NKAP promoted NB1 and SK-N-SH cell apoptosis.

### Knockdown of Nuclear Factor-κB Activating Protein Inhibited the Phosphatidylinositol 3-Kinase/Protein Kinase B Signaling Pathway in SK-N-SH Cells

**FIGURE 5:**
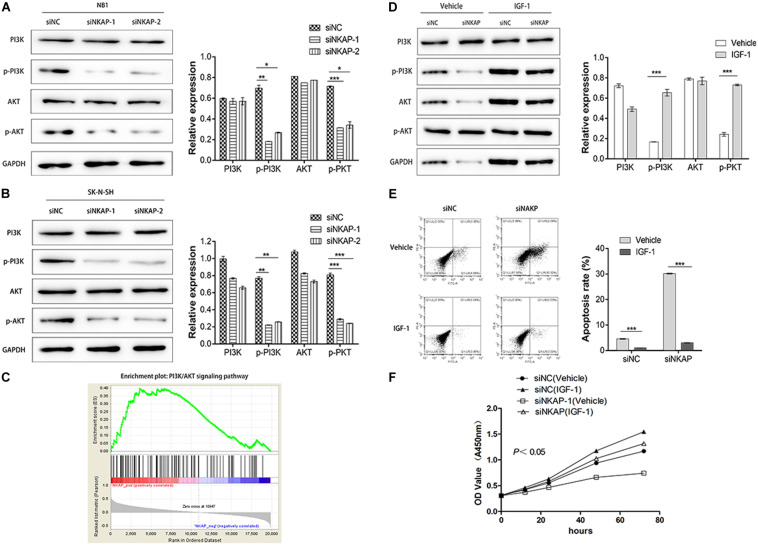
NKAP specific function in neuroblastoma cells *via* activated PI3K/AKT pathway. Transfected with siNKAP significantly reduced the phosphorylations of PI3K and AKT in NB1 **(A)** and SK-N-SH **(B)** cells *in vitro*. **(C)** Gene Set Enrichment Analysis. Activation of the PI3K/AKT pathway transfected with siNKAP did not reduce the expression of p-PI3K and p-AKT in SK-N-SH cells **(D)**, transfected with siNKAP did not promote the apoptosis of SK-N-SH cells **(E)**, and transfected with siNKAP did not inhibit the proliferation of SK-N-SH cells **(F)**. ***P < 0.001, **P < 0.01, *P < 0.05.

The PI3K/AKT signaling pathway is an important signaling pathway involved in various malignant tumors, including NB (King et al., 2015). This pathway is associated with tumor proliferation, aggressiveness, metastasis, and apoptosis. To investigate the role of NKAP in this signaling pathway, we utilized CCK-8, Western blotting, and cell apoptosis assays to verify its function. As shown in Figures 5A,B, knockdown of NKAP significantly reduced the phosphorylation of PI3K and AKT *in vitro*. Next, we used a gene set enrichment analysis (GSEA v2.2.3)^4^ to determine the enrichment score of specific signatures in the gene sets positively correlated with NKAP expression in NB profile. Samples from the NB dataset mentioned earlier were divided into high- or low-NKAP expression groups using the median as the cutoff. The results show that NKAP was positively correlated with the activation of the PI3K/AKT signaling pathway (Figure 5C). Subsequently, we activated this pathway using insulin-like growth factor 1, and Western blotting showed that siNKAP did not downregulate the expression of p-PI3K and p-AKT (Figure 5D). Cell apoptosis results showed that the apoptosis percentage of siNKAP-transfected SK-N-SH cells was significantly lower in the insulin-like growth factor 1 interference group than in the vehicle group (Figure 5E). The CCK-8 results showed that transfection with siNKAP did not reduce the proliferation of SK-N-SH cells when activating the PI3K/AKT signaling pathway (Figure 5F). Accordingly, we surmised that dysregulation of NKAP might play a significant role in the activation of the PI3K/AKT pathway in NB.

### Knockdown of Nuclear Factor-κB Activating Protein Inhibited the Phosphatidylinositol 3-Kinase/Protein Kinase B Signaling Pathway in Neuroblastoma Tumor-Bearing Models

**FIGURE 6:**
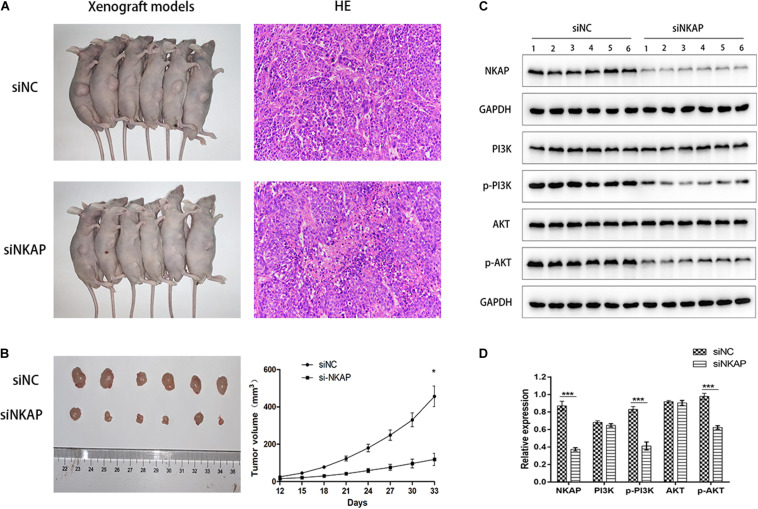
**(A)** Xenograft models inoculation with SK-N-SH cell transfected with siNKAP or siNC. **(B)** Xenograft tumors of SK-N-SH cell at 33 days after inoculation with siNKAP and siNC subcutaneously and the growth curves of xenograft models. **(C)** Function verification of NKAP in PI3K/AKT pathway by Western blot assay *in vivo*. **(D)** Quantification of NKAP function in PI3K/AKT pathway. ***P < 0.001, *P < 0.05.

We established tumor-bearing models of SK-N-SH cells to detect the expression of NKAP protein and verify its function in the PI3K/AKT pathway by Western blotting assay *in vivo*. The growth curves of tumor-bearing models transfected with siNC and siNKAP were analyzed. The tumor volume of the siNKAP models was lower than that of the siNC models (Figure 6A). The growth rate of the siNKAP tumor-bearing models was significantly faster than that of the siNC models (Figure 6B). The expression of NKAP significantly decreased in SK-N-SH models after transfection with siNKAP compared with siNC (Figures 6C,D). The function results of NKAP in the PI3K/AKT pathway were consistent with the *in vitro* results; siNKAP transfection significantly reduced the expression of p-AKT and p-PI3K compared with siNC groups.

## Discussion

NB is one of the most common cancers in children. Although advances have been made in terms of diagnostic and therapeutic methods, the 5-year survival rate of high-risk patients with NB remains below 50% (Su et al., 2020). For these high-risk patients with NB, tumor recurrence prevention is particularly difficult. Patients who experience NB recurrence may require more intensive therapy at the early stages. Therefore, it is beneficial to develop a method for predicting NB recurrence during early phases, in addition to current imaging modalities and cytological examinations. To the best of our knowledge, this is the first study to demonstrate that NKAP expression is significantly overexpressed in the recurrence group compared with the non-recurrence group. Whether NKAP is a valuable marker for predicting NB prognosis and recurrence remains to be further explored.

NKAP plays an important role in regulating human cell functions. NKAP is a nuclear-localized protein that promotes tumor necrosis factor and interleukin-1-induced NKAP activation (Chen et al., 2003). More specifically, NKAP regulates RNA splicing and processing (Burgute et al., 2014). Su et al. (2020) reported that high plasma cell-free DNA concentration is a potential molecular marker signaling disease recurrence in high-risk patients with NB. Cell-free DNA fragments contain signals from which the tissue or cellular origin of this DNA can be derived (Snyder et al., 2016). The main source of cell-free DNA included apoptotic–hematopoietic cells. In oncological patients, most cell-free DNA is formed by the apoptosis and necrosis of tumor cells (Jahr et al., 2001).

In contrast, we found that the expression of NKAP in NB tissues was related to chromosome 11q deletion. Upregulated expression of NKAP was more frequently detected with chromosome 11q deletion in the tissue, which was consistent with chromosomal instability caused by dysregulated NKAP. Chromosome 11q deficiency was more frequently observed in high-risk patients with NB (George et al., 2007; Mlakar et al., 2017). Knockdown of NKAP causes chromosome misalignment and prometaphase arrest in human cells. NKAP is critical for chromosome alignment, as it anchors CENP-E to kinetochores. It is a novel and key regulator of mitosis, and its dysregulation may contribute to tumorigenesis by causing chromosomal instability (Li et al., 2016). Srivatsan’s study demonstrated that chromosome 11q deletion is associated with advanced stage NB and poor prognosis (Srivatsan et al., 1993). Our findings indicate that NKAP may be a significant biomarker for predicting NB recurrence. We also investigated the regulatory mechanism of NKAP in NB cells both *in vivo* and *in vitro*.

NKAP is an oncogene in various malignant tumors. In glioma, NKAP can accelerate gliomas via Notch1 signaling and is considered an important oncogenic factor (Gu et al., 2019). In hepatocellular carcinoma and breast cancer, NKAP promotes the proliferation and invasion of tumor cells through the AKT/mammalian target of the rapamycin signaling pathway (Liu et al., 2018; Song et al., 2019). Therefore, we speculated that NKAP may be plays an oncogenic role in NB tumorigenesis. CCK-8 and cytometry assays showed that knockdown of NKAP significantly inhibited proliferation and promoted apoptosis of NB1 and SK-N-SH cells *in vitro*. Western blot assays showed that siNKAP decreased the expression of anti-apoptotic protein Bcl-2 and the proliferation-related proteins PCNA and Cyclin D1. Inversely, siNKAP increased the expression of pro-apoptotic proteins Bax and Caspase3. Xenograft models transfected with siNKAP inhibited SK-N-SH tumor growth. These results suggest that NKAP inhibited the proliferation and promote the apoptosis in the progression of NB.

NKAP, which functions as a transcriptional repressor, is required for iNKT cell development, T cell maturation, and the acquisition of functional competency by modulating Notch-mediated transcription as well as hematopoietic stem cell maintenance and survival (Pajerowski et al., 2009, 2010; Thapa et al., 2013). The frequency and type of iNKT and natural killer cells are associated with improved outcomes in several solid and liquid malignancies, including NB. iNKT cells and natural killer cells inhibit tumor-associated macrophages and myeloid-derived suppressor cells, kill cancer stem cells and neuroblasts and robustly secrete cytokines to recruit additional immune factors (Pajerowski et al., 2010; Nair and Dhodapkar, 2017). Notably, the production of cytokines interferon-γ and interleukin-12 by the activated iNKTs and dendritic cells results in enhanced NK- and T cell-mediated antitumor responses (Nair and Dhodapkar, 2017; King et al., 2018). Similarly, in the present study, knockdown of NKAP significantly inhibited proliferation, promoted apoptosis, and changed the prognosis of patients with NB. The interaction between NKAP and iNKT cells in the progression of NB remains to be further explored.

The PI3K/AKT pathway is a critical signaling pathway involved in various malignant tumors, and it is associated with tumor proliferation, cell migration, cellular processes, physiological functions, aggressiveness, metastasis, and apoptosis (Wagner and Nebreda, 2009; Chaturvedi et al., 2018). Recently, the PI3K/AKT pathway has been identified as a novel molecular target for cancer therapy and has the potential to improve individualized cancer therapy regimens (Polivka, and Janku, 2014). PI3K/AKT acts downstream of the vascular endothelial growth factor receptor-2 signaling pathway (Simons et al., 2016), regulates NB progression, and promotes the survival of NB stem cells after radiation (Hartmann et al., 2006; Li et al., 2019). In this study, we found that siNKAP significantly reduces the phosphorylation of PI3K and AKT both *in vitro* and *in vivo*. Accordingly, we concluded that NKAP knockdown in NB cells significantly inhibits the activation of the PI3K/AKT signaling pathway, as NKAP functions as a regulator in promoting the proliferation and inhibiting the apoptosis of NB cells by activating the PI3K/AKT signaling pathway.

## Conclusion

NKAP mediates the tumorigenesis of human NB cells by inhibited proliferation and promoted apoptosis through activating the PI3K/AKT signaling pathways, and the expression of NKAP may act as a novel biomarker for predicting recurrence and chromosome 11q deletion in patients with NB.

## Data Availability Statement

The datasets generated for this study can be found in the online repositories. The names of the repository/repositories and accession number(s) can be found in the article/supplementary material.

## Ethics Statement

This study was approved by the Research Ethics Committee of Beijing Friendship Hospital, Capital Medical University (IRB: 2018-P2-145-02). The patients/participants provided their written informed consent to participate in this study. All animal experiments were in compliance with the protocols approved by the Institutional Animal Care and Use Committee (IACUC) of the Institute of Medicinal Biotechnology, Chinese Academy of Medical Sciences (IMBF20200301).

## Author Contributions

JY and JG contributed to conception and design. JuL and MZ contributed to the acquisition of data or analysis and interpretation of data. JuL, MZ, YK, WW, JiL, JG, and JY performed the experiments. JuL, MZ, JY, and JG have been involved in drafting the manuscript or revising it critically for important intellectual content. All authors have given final approval of the version to be published.

## Conflict of Interest

The authors declare that the research was conducted in the absence of any commercial or financial relationships that could be construed as a potential conflict of interest.

## Footnotes

1 https://www.ebi.ac.uk/arrayexpress/experiments/E-MTAB-8248/

2 http://software.broadinstitute.org/gsea/index.jsp

3 http://software.broadinstitute.org/gsea/msigdb/collections.jsp#H

4 http://software.broadinstitute.org/gsea/index.jsp
